# Conducting a pediatric randomized clinical trial during a pandemic: A shift to virtual procedures

**DOI:** 10.1017/cts.2022.453

**Published:** 2022-08-25

**Authors:** James R. Roberts, Sheva K. Chervinskiy, Russell McCulloh, Jessica Snowden, Paul M. Darden, Thao-Ly T. Phan, Erin Dawley, Victoria Reynolds, Crystal S. Lim, Lee Pyles, DeAnn Hubberd, Jaime Baldner, Lora Lawrence, Ann M. Davis

**Affiliations:** 1 Medical University of South Carolina, Charleston, SC, USA; 2 University of Arkansas for Medical Sciences, Little Rock, AR, USA; 3 University of Nebraska Medical Center, Omaha, NE, USA; 4 University of Oklahoma Health Sciences Center, Oklahoma City, OK, USA; 5 Nemours Children’s Health, Wilmington, DE, USA; 6 West Virginia University, Morgantown, WV, USA; 7 University of Mississippi Medical Center, Jackson, MS, USA; 8 Center for Children’s Healthy Lifestyles & Nutrition, Kansas City, MO, USA; 9 University of Kansas Medical Center, Kansas City, KS, USA

**Keywords:** Pediatric, rural, remote clinical trials, decentralized clinical trials, obesity

## Abstract

**Background/Objective:**

Prior to the COVID-19 pandemic, our research group initiated a pediatric practice-based randomized trial for the treatment of childhood obesity in rural communities. Approximately 6 weeks into the originally planned 10-week enrollment period, the trial was forced to pause all study activity due to the COVID-19 pandemic. This pause necessitated a substantial revision in recruitment, enrollment, and other study methods in order to complete the trial using virtual procedures. This descriptive paper outlines methods used to recruit, enroll, and manage clinical trial participants with technology to obtain informed consent, obtain height and weight measurements by video, and maintain participant engagement throughout the duration of the trial.

**Methods:**

The study team reviewed the IRB records, protocol team meeting minutes and records, and surveyed the site teams to document the impact of the COVID-19 shift to virtual procedures on the study. The IRB approved study changes allowed for flexibility between clinical sites given variations in site resources, which was key to success of the implementation.

**Results:**

All study sites faced a variety of logistical challenges unique to their location yet successfully recruited the required number of patients for the trial. Ultimately, virtual procedures enhanced our ability to establish relationships with participants who were previously beyond our reach, but presented several challenges and required additional resources.

**Conclusion:**

Lessons learned from this study can assist other study groups in navigating challenges, especially when recruiting and implementing studies with rural and underserved populations or during challenging events like the pandemic.

## Introduction

Children with obesity represent a large and understudied population that is at increased risk of health conditions that may follow them into adulthood [1]. Children in rural areas are disproportionally affected with obesity and are underrepresented in clinical trials [2-4]. Recruiting children and families in rural locations to participate in studies can present logistical challenges, particularly related to limited accessibility including extra time and travel for rural participants [4,5]. Engaging local medical providers and clinics, such as through a practice-based research network, has been shown to increase participation among rural families [6]. Factors that enhance recruitment of rural participants into pediatric obesity clinical trials include providing monetary time incentives, cultivating relationships with clinics, emphasizing potential benefits to participants, and focusing on healthy lifestyle rather than on obesity [7,8].

Prior to the COVID-19 pandemic, clinical trials were primarily conducted through face-to-face communication. Research coordinators engaged participants via in-person contact for study procedures such as recruiting, consenting, enrolling, and performing study visits. However, in early 2020, the COVID-19 pandemic caused local and state governments to mandate that people stay at home and/or have limited in-person contact causing most clinical trials to initially halt study activities [9]. When assessing how to safely resume research activity, study teams explored the use of technology and created innovative ways to overcome obstacles. The use of technology, particularly telemedicine tools, provides an opportunity for a positive change in the recruitment and conduct of clinical trials [10,11].

Pre-pandemic our team set out to recruit and enroll rural participants into the *Feasibility Trial of the iAmHealthy Intervention for Healthy Weight in Rural Children Recruited from Primary Care Clinics* (*iAmHealthy*; NCT04142034) [12]. This trial was based on a similar study that was conducted in schools [2]. The current pediatric practice-based randomized trial is a feasibility study aimed at demonstrating that overweight pediatric participants in a rural setting could be successfully recruited to a clinical trial of a technology-based lifestyle behavioral intervention. Enrollment began on February 1, 2020. Approximately 6 weeks into the 10-week enrollment period, the trial was forced to pause all study activity due to the COVID-19 pandemic. While paused, the study team worked to develop a process by which the clinical sites could transition to a virtual approach to complete all aspects of the trial safely and effectively.

The purpose of this paper is to describe real-time experiences in the field and present the challenges that each site overcame to reach its goals and the processes they used to do so. Many of the experiences from this study can inform future clinical trials aimed at populations facing geographic or institutional challenges to in-person participant contact. These methods of recruitment, consent, and implementing the study may be useful in newer studies to help facilitate virtual recruitment, especially in groups historically hard to recruit.

## Methods

The methods of the clinical trial are described elsewhere [12]. Briefly, the trial was focused on recruiting up to 128 participants aged 6–12 years of age who reside in rural regions in one clinic in each of four states (DE, NE, SC, and WV). Each clinic was selected by their states’ awardee site which are members of the Environmental Child Health Outcomes (ECHO) IDeA States Pediatric Clinical Trials Network (ISPCTN) [13]. Criteria for the clinic’s participation included having 40% of pediatric patient visits covered by Medicaid, having seen a minimum of 100 potential patients (rural, 6–11 years of age, and overweight or obese) within the past year, and having appropriate resources to recruit and enroll participants. For a full list of criteria see Davis et al., 2021 [12]. The rural criteria was based on having at least 50% of patients residing in a ZIP code area classified by rural-urban commuter area (RUCA) codes 4 or higher as a measure of rurality [14] (https://www.ers.usda.gov/data-products/rural-urban-commuting-area-codes/, accessed August 3, 2022). The current trial randomized four clinical sites to start with either “consecutive” recruitment or “traditional” recruitment periods of one month each. This was followed by a switch to the other recruitment method for 4 weeks and then a planned 2-week make-up recruitment period with the method that worked best for each site, for a total recruitment of 10 weeks. Consecutive recruitment is defined by using a list of patients recently seen in the clinic (or upcoming visits) who meet study criteria. Research staff actively contact the patients/parents to ask them if they would like to participate in the study. Traditional recruitment methods include passive methods such as posters or fliers that are posted in the clinic space with a phone number for interested families to contact, as well as referral by the health care provider to the study team. Once potential participants were identified, enrollment consisted of informed consent of parent, assent of child, and height and weight measurements of the child [12].

We reviewed four data sources to describe the transition to virtual study procedures. The first included responses to a research electronic data capture (REDCap) survey administered prior to resuming study procedures during the COVID-19 pandemic, completed by the principal investigator (PI) and research coordinator at each of the four sites [15]. The 26-item survey assessed site readiness to transition to all virtual procedures and included questions about the status of in-person research and clinical activities at the site, feasibility, and methods to be used for obtaining consent and anthropometric measurements, accessing medical records and obtaining other supplies needed to conduct research, and feasibility of communicating with participants and providers at the clinic.

The second source of data included meeting minutes, maintained by the ISPCTN data coordination and operation center (DCOC), from weekly meetings attended by the study protocol chairs, site PIs, site research coordinators, and DCOC project managers during study implementation. These hour-long meetings focused on the status of recruitment, enrollment, and data collection at each site, as well as discussion of challenges encountered by sites during study implementation.

A third source of data included responses to an electronic REDCap survey administered upon completion of the study to each site team (site PI and research coordinators). The survey consisted of seven open-ended questions, assessing changes each site made to adjust to COVID-19 restrictions, barriers to implementing these changes, and opportunities that these changes provided in the following areas: screening, recruitment, consent, baseline height and weight measurements, issues with shipping of study materials (such as activity monitors), data collection, and participant payments.

Finally, enrollment data were collected by the DCOC in REDCap during the study. For the purposes of this manuscript, enrollment data are described based on timing of enrollment (before and after the pause in study activities due to the pandemic). Full details about study enrollment will be reported elsewhere. The iAmHealthy Feasibility trial and all study modifications associated with the shift to virtual procedures were approved by the UAMS IRB, which serves as the single IRB (sIRB) for this study.

## Results

### Recruitment/Screening for Study Eligibility

At the time of the study pause, 6 of the planned 10 weeks of recruitment had been completed, with 42 subjects total having been consented and one screening failure and one early termination. All study activities resumed after 12 weeks with the same recruitment methods still in place. Sites completed the final two weeks of the recruitment method the site was utilizing at the time of the study pause. The study team extended the catch-up enrollment period from 2 weeks to 4 weeks, to account for the loss of participants who could not be re-engaged following the study pause and to accommodate study procedure changes. Note that there was 6 weeks of enrollment prior to the 12-week pause followed by 6 weeks of enrollment after the pause for a total enrollment period of 12 weeks. The net increase in actual study time compared to the original protocol was the additional 2 weeks in the catch-up recruitment period and 4 weeks in the post assessment period to allow for shipping of materials.

As detailed in Table 1, a total of 117 participants were recruited for the study, including 28 who re-consented and 89 new participants who consented for the virtual study procedures following the study pause. A total of 42 participants were consented prior to the study pause; however, fourteen participants were not successfully re-engaged after the study pause despite numerous attempts by the study team. Much of this was related to an enormous social disruption during COVID, with many families losing their jobs, disconnected phones, and moving in with other family, and far from their original primary care provider’s office.

**Table 1.** Numbers of participants recruited by clinical site (state)

Pre-COVID Pause in Study Activity		Post-COVID Pause in Study Activity		
	In-person consent	Re-consented participants*	New recruited/consented participants*	Total**
Delaware	5	5	24	29
Nebraska	5	4	29	33
South Carolina	18	14	18	32
West Virginia	14	5	18	23
Total	42	28	89	117

* Recruitment and informed consent using virtual technology.

** Total does not reflect participants who did not re-consent.

### Consent/Re-Consent

At the beginning of study activities, the protocol dictated informed consent be obtained in person. During the pause in study activities, the protocol was amended to allow for consent via virtual procedures. To afford the study sites maximum flexibility in communicating with study participants, the protocol was expanded to allow IRB-approved study documents to be sent to the participants via hand delivery, postal mail, email, fax, REDCap, or other electronic platforms. The coordinators were allowed to discuss the study with the participants over the telephone or using video conferencing platforms, depending on the tools and guidelines at the site.

There were multiple options for consent/reconsent based on the type of electronic platforms available at each institution. Once the participant agreed to proceed with study activities, options for returning signed consent documents to the research coordinator included: 1) handing them back, 2) mailing with a wet signature, 3) scan and fax, 4) text or email a picture of the signed signature page, or 5) type the signature and date into the electronic version of the consent and electronically return to with a note stating that this is the legal authorized representative’s electronic signature. Lastly, if these methods were not available to the participants, the participants could send a well-worded email back to the coordinator that stated the name of the study and that the participant read and discussed the document and gave consent to participate in the study.

Similar procedures were used to obtain assent when applicable. Additionally, the person obtaining consent documented the consent process including the method of consent, questions asked, people involved in the conversation, and other details of the consent process. In addition, at the time of the study pause and the new procedures were developed, a new consent document was required, thus all previously enrolled participants were reconsented. HIPAA authorization forms were signed using the same virtual procedures.

These changes provided many opportunities for enhanced enrollment of participants and a few challenges (Table 2). Overall, these changes were more convenient for the study team and participants. Consent visits were able to occur at any time, whereas previously they were reliant on the hours of the primary care clinic where recruitment occurred. The study team and participants also no longer needed to travel to the rural clinic site for consent visits and there was flexibility in scheduling and rescheduling consent visits if needed. Finally, for those sites that used an electronic platform to obtain consent, this added an additional layer of electronic tracking of participants that was useful. However, there were some participants who had technical challenges (slow internet, difficulties understanding electronic consent forms) which extended the time needed for those participants to complete the consent process.

**Table 2.** Summary of the opportunities and barriers that were encountered with changes to a virtual study approach

Opportunities	Study team was able to conduct study visits (including recruitment, consent, and screening visits) at any time of the day via virtual methods A participant that “no showed” to an appointment did not impact travel to a rural site for the study team Participants and study team did not have to travel to the clinical site for study visits Participants were able to keep the weight scale and other equipment Two sites were able to collect height and weight measurements at the end of the study by both in-person and virtual methods, in order to validate the virtual measurements
Challenges	Limited Internet access and/or slow Internet speeds in rural areas, which limited participant engagement and decreased the time for the e-consent process Some participants had computers without a camera, so that they were unable to continue the study virtually A second visit was required to screen patients due to having to ship screening materials Study team had to rely on participants to take their own height and weight measurements Reliance on external delivery service for study related materials, including screening materials and activity monitors resulted in delays to conducting the enrollment visit Depending on the platform being used, there were delays in uploading study documents Increased study costs to pay for postage for additional shipments, additional study materials, and for some families Internet-connected tablets for electronic consenting

### Virtual Height and Weight Assessment

Prior to the study pause, in-person screening visits were completed at the participating clinics using research grade equipment provided by the study to validate the child’s eligibility for the trial based on BMI percentile for age. Consent and screening were obtained at the same visit.

After the pause, height and weight assessment were completed as a separate virtual visit from the consent/assent visit. Study teams from each site shipped materials to consented participants. These supplies included a digital scale, measuring tape, painter’s tape, and ruler to facilitate virtual height and weight screening assessments with participating families. Once the family obtained the equipment, a video visit (by FaceTime or other institution approved platform, such as Doxy.me) was conducted with trained staff members guiding parents to obtain the child’s height and weight while observing by video to ensure accuracy. The height and weight procedures used in the study were adapted from another ongoing project being conducted by the investigator team (NCT03304249). The same height and weight procedures were used for the post-intervention measurements 6 months after the baseline measurements. Two of the sites in the study obtained both in-person and virtual height and weight measurements at the end of the study, which will allow future validation of the virtual height and weight procedures used in this study.

If a nonqualifying BMI percentile was obtained during the virtual screening visit, then the family was asked to return the equipment to the site. All qualifying participants were able to keep their height and weight study materials through the duration of the study for use during the final assessment and after the completion of the trial.

As with recruitment and consent, the shift to virtual procedures allowed study teams to be flexible in their communications with participants (Table 2). Study teams employed various means of electronic communication to schedule screening visits and interact with participants including text messaging on cellphones, video conferencing, and emailing. The study teams also offered extended hours for participant contact with the study team. However, these protocol changes did present some logistical challenges. For example, the changes added 2 weeks to the screening period to allow for shipping of the materials. Some study participants only provided a PO Box address, whereas courier services required a physical street address adding to shipping delays. Finally, some participants had difficulty reading the tape measure in order to obtain accurate heights and needed additional guidance and validation from the study team over video to complete the measurements.

### Activity Monitor Data

Prior to the pause in study activities, study participants were given activity monitors to wear for 7 days to track their physical activity during their in-person consent visits. The activity monitor was initiated to start tracking the participant’s activity immediately. While in-person, the study team was able to demonstrate and instruct the participant on how to properly wear and care for the activity monitor. After shifting to virtual procedures, activity monitors were mailed out with a return envelope via USPS or other courier service. All instructions on wearing the activity monitor were discussed on the virtual video screening visit or by phone.

Overall, the shift to virtual procedures provided challenges with regard to the collection of activity monitor data (Table 2). For example, the initiation date of the activity monitor had to account for shipping time. Shipping times varied across sites and not only impacted the study team sending activity monitors to participants but also participants sending activity monitors back to the study team. If the participant had to re-wear the activity monitor due to insufficient wear time on their first attempt, this resulted in additional time and shipping costs, or in some instances, additional costs and time due to hand delivery by the study team. Finally, software to download activity monitor data needed to be transferred to devices that study teams were using.

### Financial/Cost Considerations

There were several financial changes to consider in the transition to virtual procedures. Across all sites, study costs were increased by (1) measurement tools for each participant, (2) shipping costs associated with the measurement tools and activity monitors, and (3) internet-enabled tablets for participants in the control group who did not have their own video-enabled device for virtual assessments. Some of these costs were offset by a decrease in travel costs for research teams and participants to the clinical site. Unanticipated costs for lost or damaged equipment also need to be considered. Rather than taking possession of the Internet-enabled tablets and activity monitors prior to issuing the final payment, we relied on families to return the tablets and activity monitors through the US mail. Of the 66 tablets distributed, 15 were not returned, and 7 were damaged upon receipt. In addition, 28 activity monitors were not returned. Overall, we estimate a 20–30% increase in costs related to the shift to virtual procedures but this is approximate given the many factors affecting budgeting and invoicing.

At the individual site level, shifting to virtual participant reimbursement also highlighted the need to adjust regulatory processes around payments. Because payments were distributed via mail rather than on-site, site teams, and the coordinating center adjusted processes for tracking participant engagement and triggers to distribute repeated payments with each study visit. Sites also had to work with their local finance offices to document participant receipt of payments within institutional guidelines.

When transitioning a clinical trial from on-site to virtual procedures, it is important to consider the significant added costs and effort that can come from additional participant equipment that will be needed, shipping costs, technical support for participants for home procedures, additional instructional materials for families related to home procedures, and adjusting processes to document participant payments.

## Discussion

The COVID-19 global pandemic led to paradigm shifts in all scientific sectors. While iAmHealthy is not the first, nor the only, clinical trial that needed to undergo a rapid transformation for recruitment, few studies to date have published the challenges of recruiting. Some studies describe the use of digital technology in trials prior to the pandemic [16-18]. Other sources present a theoretical basis of how digital clinical trials or telehealth-based research may be accomplished based on current clinical uses of digital technology [19-22]. Here we describe procedural changes with regard to recruitment, screening, data collection, and regulatory study activities in a multicenter clinical trial focused on pediatric rural populations during the COVID-19 global pandemic. The lessons learned from the iAmHealthy experience highlight the importance of flexibility in study procedures; understanding the regulatory considerations with a shift to virtual procedures; and committing added financial support to successfully complete clinical trials in children in hard-to-reach areas.

Rural pediatric populations are underrepresented in scientific research due to procedural and psychological barriers to carrying out clinical trials in these areas. These challenges include limited accessibility of participants, misperceptions about clinical research, and distrust of scientific establishments [23]. With the goal of addressing these barriers, the initial design of the iAmHealthy study was aimed at enhancing recruitment of rural trial participants through the engagement of local clinics and highlighting study benefits. Despite the protocol changes to incorporate appropriate safety measures for the participants and researchers, this study achieved the recruitment goal.

Flexibility was key in implementation of virtual procedures given the variations in site resources. For example, the process to obtain participant consent involved various platforms at each site and the IRB approved study procedures allowed for this variation. This allowed study procedures to resume with slightly different processes based on specific conditions at each site, while the overall study remained agile and achieved the enrollment objectives. Being able to call and text potential participants from the recruitment list, as well as speak to them about the study at hours that were convenient for them instead of during a medical visit at the rural clinic was very helpful. Some sites also struggled with participant payments due to changing from paper payments to electronic payment methods while other sites had established electronic payment systems. To establish consistency in the study height and weight outcomes, all sites were required to obtain virtual measurements, but those allowed to have in person visits also obtained face-to-face measurements to validate the virtual assessments. Protocol teams should carefully consider study changes needed to incorporate additional time and flexibility in consent and assessment methods to achieve the desired study outcomes and performance standards.

Our study teams found that the virtual process of recruitment with increased flexibility of communication and variable study hours led to a higher number of recruited participants than the methods employed prior to the pandemic. It is often challenging to conduct any clinical trial or study in a rural population, but these virtual methods demonstrate the feasibility of doing this in an often difficult to reach rural population.

Successfully adapting this clinical trial to employ virtual data collection and study procedures in a rural area presented many challenges and opportunities. The lack of infrastructure in rural areas presents challenges in the timely delivery to and return of study materials from participant homes due to inconsistent mail delivery. We also noted that in this study, when compared to previous studies carried out in a school setting, we had a lower rate of return of study materials to the data coordinating center upon completion of study activities. This may have been related to transportation barriers or distance from participants’ home to the post office, or not having an in-person visit at the end of the study. Another possibility is that the study procedures did not include typical activities like home visits to retrieve equipment which may have been problematic. Similarly, several of our participants in rural areas required the study team to provide internet-enabled devices given the lack of technology in the home. Future studies utilizing technology should keep these additional time and costs in mind for planning and budgetary purposes.

## Conclusion

Despite the challenges of the pandemic, the shift to virtual methods allowed us to fulfill the recruitment requirements for the study. This shift to virtual procedures highlighted many important lessons learned. As previously noted, rural populations can be particularly challenging to engage in scientific research. In this study, we learned that virtual procedures enhanced our ability to establish relationships with participants who were previously beyond our reach. Primarily, we found that agility and flexibility of the study team and the clinic sites were key factors in maintaining integrity of study activities. Adaptability in scientific study procedures is one of the keys to successfully completing research during challenging times. Ideally lessons learned from this study will assist other study groups in navigating challenges, especially when recruiting in rural populations.
